# Evaluating the Inflammatory Protein Ratio (IPR) as an Inflammation-Based Biomarker for Cancer Diagnosis

**DOI:** 10.3390/ijms26094375

**Published:** 2025-05-05

**Authors:** Aurelio Lo Buglio, Francesco Bellanti, Rosanna Maria Carapellese, Rosanna Villani, Moris Sangineto, Antonino Davide Romano, Gianluigi Vendemiale, Gaetano Serviddio

**Affiliations:** Department of Medical and Surgical Sciences, University of Foggia, 71122 Foggia, Italy; francesco.bellanti@unifg.it (F.B.); rosanna_carapellese.549375@unifg.it (R.M.C.); rosanna.villani@unifg.it (R.V.); moris.sangineto@unifg.it (M.S.); antonino.romano@unifg.it (A.D.R.); gaetano.serviddio@unifg.it (G.S.)

**Keywords:** inflammatory protein ratio (IPR), active cancer, screening tools, inflammatory markers

## Abstract

Chronic inflammation is increasingly recognized as a key driver of tumorigenesis, affecting both the tumor microenvironment and host response. In this context, circulating inflammatory proteins may provide valuable insights into cancer activity. This study evaluated the diagnostic performance of the inflammatory protein ratio (IPR), a composite index derived from serum protein electrophoresis, in detecting active cancer among hospitalized patients. We retrospectively analyzed clinical and laboratory data from 312 adult patients admitted to the Internal Medicine and Aging Department at Policlinico Foggia, Italy, between November 2023 and July 2024. Patients were stratified according to the presence of active cancer, defined by NICE criteria. The diagnostic accuracy of the IPR was compared with that of conventional inflammatory markers, including C-reactive protein (CRP) and the neutrophil–lymphocyte ratio (NLR), platelet–lymphocyte ratio (PLR), monocyte–lymphocyte ratio (MLR), and systemic immune–inflammation index (SII). The IPR showed the highest diagnostic performance, with a sensitivity of 88.1%, a specificity of 75.2%, and an area under the receiver operating characteristic curve (AUC) of 0.868. Its negative predictive value reached 97.6%, underscoring its potential as a rule-out tool for malignancy in hospitalized patients. These findings support the IPR as a promising and cost-effective inflammation-based biomarker for cancer detection, warranting further validation in prospective and molecularly characterized cohorts.

## 1. Introduction

Cancer remains a major contributor to global disease burden with substantial health, social, and economic impacts [1]. In 2022 alone, an estimated 20 million new cancer diagnosed recorded globally, resulting in nearly 9.7 million deaths [2]. The incidence and mortality of cancer exhibit wide geographic variability, reflecting differences in healthcare access, risk factor distribution, and early detection programs [3,4]. Projections indicate a steady rise in new cancer cases, underscoring the importance of early detection to improve clinical outcomes and reduce healthcare costs [2,5].

The prevalence of cancer among hospitalized patients has steadily increased, placing additional demands on healthcare systems [6]. These patients often present with complex clinical needs, including prolonged hospital stay and higher in-hospital mortality [7,8]. Conditions such as immunosuppression, sarcopenia, malnutrition, and prior infections have been identified as relevant contributors to adverse outcomes in this population [9,10]. 

Chronic low-grade inflammation contributes to multiple stages of tumor development, including cell proliferation, angiogenesis, tissue invasion, and metastatic progression [11,12,13]. The tumor microenvironment drives the release of a wide range of cytokines, and the specific cytokine profiles can differ substantially depending on the tumor type and disease stage. For example, in renal cell carcinoma, elevated IL-6 levels are linked to metastasis, while in breast cancer, distinct cytokine signatures have been shown to discriminate between tumor subtypes and predict metastatic potential. These findings underscore that even within a single type of cancer, such as breast cancer, the inflammatory cytokine profile can vary according to biological differences among tumor subtypes [14]. Cytokines also play central roles in the pathogenesis of chronic inflammatory and autoimmune diseases, such as rheumatoid arthritis, inflammatory bowel disease, and psoriasis, further highlighting their relevance across a wide spectrum of immune-mediated conditions [15,16]. Inflammation at diagnosis may have significant prognostic implications. Higher degrees of systemic or local inflammation are associated both with increased risk of tumor progression and with reduced overall survival. Several studies have shown that chronic inflammation not only facilitates tumor growth and metastasis but can also impair the host immune response, leading to worse clinical outcomes. Moreover, emerging evidence suggests that the inflammatory profile at baseline may predict the response to anticancer therapies. Given that inflammatory pathways are often genetically stable and less prone to rapid mutation compared to tumor cells, targeting cancer-associated inflammation is now considered a promising therapeutic strategy to improve outcomes and overcome treatment resistance [13,17]. The interplay between systemic inflammation and tumor biology has prompted interest in identifying blood-based biomarkers that may aid in early cancer detection and prognostication [18,19]. Several indices based on peripheral blood cell ratios have been proposed and investigated, including the neutrophil-to-lymphocyte ratio (NLR), platelet-to-lymphocyte ratio (PLR), monocyte-to-lymphocyte ratio (MLR), and the systemic immune-inflammation index (SII). However, these inflammatory markers have demonstrated only moderate diagnostic accuracy in detecting cancer [20,21,22]. In parallel, serum proteins such as albumin, α1-globulin, and α2-globulin—measured through serum protein electrophoresis (SPE)—are well-established markers of systemic inflammation, and alterations in their levels have been associated with malignancy [23,24,25]. Recently, Antonucci et al. proposed the Inflammatory Protein Ratio (IPR), a composite score derived from these protein fractions, initially designed to monitor inflammation in COVID-19 patients [26].

This study aims to evaluate the diagnostic ability of the IPR in identifying active cancer in hospitalized patients and to compare its performance with established inflammation-based markers. 

## 2. Results

### 2.1. Baseline Characteristics

A total of 312 patients were retrospectively recruited, of whom 163 (52.2%) were female. The median age was 70.0 years [59.2–81.0]. A total of 42 (13.5%) patients had an active cancer. Thoracic and digestive tumors were the most prevalent types of cancer among our population. The type of cancer and its prevalence according to the 5th edition of WHO classification are reported in Table 1 [27]. No significant differences were found between the two groups in terms of age, gender, monocyte count, or creatinine levels. Haemoglobin, albumin, and lymphocyte counts were lower in the cancer group compared to the control group. WBC, neutrophil counts, platelet counts, CRP, IPR, NLR, PLR, MLR, and SII were higher in the cancer group compared to the control group. The data are summarized in Table 2.

### 2.2. Diagnostic Value of IPR Versus Other Pro-Inflammatory Indices in Identifying the Presence of Cancer

**Figure 1:**
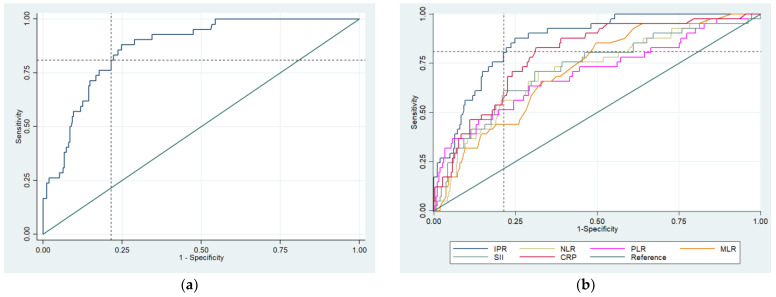
The diagnostic value by receiver operator characteristics curves (**a**) of IPR and (**b**) IPR, CRP, NLR, PLR, MLR, and SII. Abbreviation: IPR, inflammatory protein ratio; CRP, C-reactive protein; NLR, neutrophil–lymphocyte ratio; PLR, platelet–lymphocyte ratio; MLR, monocyte–lymphocyte ratio; SII, systemic immune–inflammation index.

**Figure 2:**
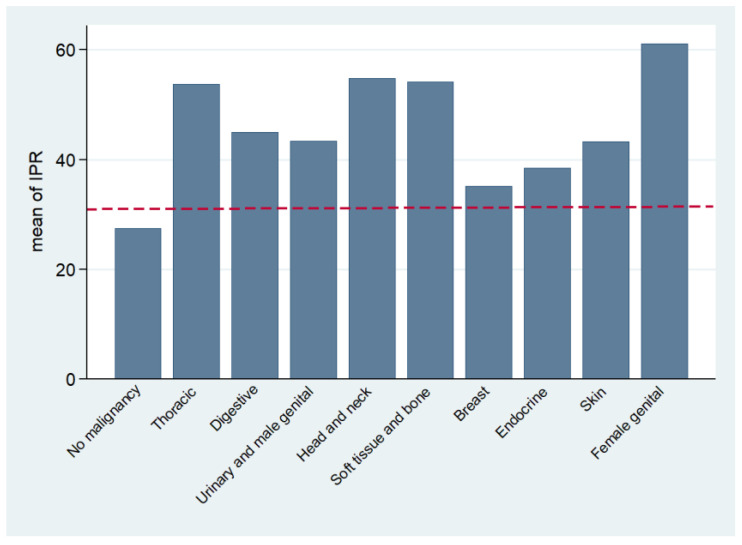
Bar graph displaying the IPR cutoff value in relation to the average IPR values for various cancer types versus the control group. Abbreviations: IPR, inflammatory protein ratio.

Table 3 summarizes the diagnostic performance of IPR, CRP, NLR, PLR, MLR, and SII. The analysis of diagnostic performance showed that IPR, with a cut-off of ≥31, had the highest sensitivity (88.1%) and specificity (75.2%), with a positive likelihood ratio (+LR) of 3.55 and a negative likelihood ratio (−LR) of 0.16, indicating its strong ability to differentiate between cases. IPR also had the highest area under the curve (AUC = 0.868) and a positive predictive value (PPV) of 35.7%, with a negative predictive value (NPV) of 97.6%, highlighting its diagnostic usefulness (Table 3, Figure 1a). Figure 2 shows the distribution of IPR values by cancer type compared to controls, including the identified cutoff. CRP showed a sensitivity of 82.9% and specificity of 68.4%, and an AUC of 0.790. NLR, PLR, MLR, and SII demonstrated lower sensitivities and AUCs, but all showed statistically significant results (*p* < 0.001) (Table 3, Figure 1b). These results indicate that IPR provides superior diagnostic accuracy compared to other inflammatory indices.

Interestingly, the comparison of AUC presented in Table 4, confirmed that IPR performed better with statistically significant differences compared to all other markers. The pairwise comparison revealed that PLR had the largest AUC difference compared to IPR, followed by NLR and MLR. CRP and SII exhibited smaller AUC differences when compared to IPR.

### 2.3. Comparative Diagnostic Value of IPR Based on Reclassification Metrics

To further assess the incremental diagnostic value of the IPR over conventional inflammatory markers, we calculated continuous net reclassification improvement (NRI) and its components (NRI+ and NRI−) as well as integrated discrimination improvement (IDI) for each pairwise comparison. When IPR was compared with CRP, the total NRI was 0.91 (*p* < 0.001), mainly driven by a large NRI− of 0.66, reflecting substantial improvement in the correct classification of patients without cancer. The NRI+ for CRP was 0.25, and the corresponding IDI was 0.13 (*p* < 0.001).

In comparison with NLR, the total NRI was 0.77 (*p* < 0.001; NRI−: 0.62, NRI+: 0.15) and the IDI was 0.10 (*p* < 0.001). For PLR, the NRI was 0.95 (*p* < 0.001; NRI−: 0.70, NRI+: 0.25) and the IDI was 0.12 (*p* < 0.001). In comparison with MLR, the NRI was 0.83 (*p* < 0.001; NRI−: 0.73, NRI+: 0.10) with an IDI of 0.09, while for SII, the NRI was 0.89 (*p* < 0.001; NRI−: 0.69, NRI+: 0.20) and the IDI was 0.11 (*p* < 0.001).

## 3. Discussion

The principal finding of this study is that the IPR, a novel inflammation-based biomarker, demonstrated superior diagnostic performance compared to conventional indices such as CRP, NLR, PLR, MLR, and SII in identifying active cancer. With a cut-off value of ≥31, the IPR achieved the highest sensitivity (88.1%) and specificity (75.2%), along with an AUC of 0.868, underscoring its potential as a reliable and accurate tool in this context. The high negative predictive value (NPV) of 97.6% further supports its role in safely excluding cancer in hospitalized patients, potentially reducing unnecessary diagnostic investigations. In addition to standard accuracy metrics, we assessed the incremental diagnostic value of the IPR over each conventional inflammatory marker using reclassification-based metrics. The IPR consistently demonstrated significant improvements in patient classification and risk discrimination compared to CRP, NLR, PLR, MLR, and SII. Across all comparisons, the Net Reclassification Improvement (NRI) ranged from 0.77 to 0.95, with the highest gain observed against PLR. The Integrated Discrimination Improvement (IDI) values ranged from 0.09 to 0.13, further confirming that the IPR enhances the ability to distinguish between cancer and non-cancer cases. These improvements were statistically significant in all cases (*p* < 0.01), underscoring the robustness of the IPR across various inflammatory profiles. Notably, the strongest contributions to NRI came from improved classification of patients without cancer, reinforcing the potential of the IPR as an effective rule-out tool in hospitalized settings.

Inflammation is increasingly recognized as a key enabler of cancer development and progression. Chronic immune activation—primarily driven by macrophages and other innate immune cells—can promote tumorigenesis through the release of cytokines, chemokines, and reactive oxygen/nitrogen species, creating a tissue microenvironment conducive to metaplasia, dysplasia, and ultimately malignant transformation [9,13]. Certain tumor types, including renal cell carcinoma, pancreatic neuroendocrine tumors, and hepatocellular carcinoma, have been shown to enhance systemic CRP levels indirectly, through tumor-derived production of pro-inflammatory cytokines such as interleukin-6 (IL-6), which in turn stimulates hepatic CRP synthesis [28]. Also, increasing evidence suggests that the degree of systemic inflammation in cancer patients may be influenced by the tumor burden. In particular, higher tumor load has been associated with elevated levels of inflammatory biomarkers such as IL-6, ferritin, and IL-8, reflecting an enhanced systemic inflammatory response [29]. Numerous biomarkers have been investigated to capture this cancer-related inflammatory state, including CRP, NLR, PLR, MLR, and SII, which have shown prognostic utility across different malignancies and disease stages [13,30,31,32,33,34,35,36]. Recently, interest has shifted toward exploring these markers also for diagnostic purposes, particularly in early-stage disease or in patients with non-specific clinical presentations [37]. The diagnostic potential of blood-based inflammatory markers has gained ground in recent years. CRP levels have been shown to correlate with cancer burden and disease progression, although their diagnostic interpretation requires caution, as levels are often affected by comorbidities and infection status [38]. Karra et al. demonstrated that NLR, PLR, and MLR have good diagnostic accuracy in predicting gastric cancer, suggesting their potential as screening tools in selected populations [20]. Fang et al. also reported that NLR and PLR outperformed traditional tumor markers such as CEA and CA 19-9 in the diagnosis of gastric cancer, reinforcing the link between systemic inflammation and neoplastic processes [39]. Similarly, SII has been associated with increased risk of developing solid tumors and has shown diagnostic relevance in malignancies such as thyroid and lung cancer [40,41,42]. Consistent with these findings, our results indicate that the IPR offers superior diagnostic accuracy compared to these established indices. Unlike ratios focused on circulating blood cells (such as NLR, PLR, MLR, and SII), the IPR integrates dynamic alterations in serum protein fractions that are more directly influenced by the hepatic acute-phase response, which is tightly regulated by inflammatory cytokines such as IL-6 [43,44]. These cytokines are often upregulated in malignancy and contribute to cancer-related systemic inflammation [45]. This dual behaviour makes the IPR more sensitive to chronic inflammatory signals that might be overlooked by haematological ratios alone. Specifically, albumin levels tend to decrease in the setting of inflammation due to reduced hepatic synthesis, while α1- and α2-globulins typically rise as part of the acute-phase response [26,46,47]. By integrating these parameters, the IPR provides a more nuanced assessment of systemic inflammation, potentially reflecting tumor-associated immunologic activity more precisely than cell-based ratios. 

A notable strength of our study is that it represents, to our knowledge, the first attempt to evaluate the diagnostic utility of the IPR for cancer detection in a hospitalized population. Despite the heterogeneity in tumor types and stages, including thoracic, digestive, and other cancers, the IPR maintained consistent performance, suggesting broad applicability across different neoplastic entities.

Several limitations should be acknowledged. First, the retrospective and monocentric nature of the study may limit the generalizability of our results. Furthermore, treatment-related factors such as ongoing chemotherapy or corticosteroid use were not available and could have influenced inflammatory profiles. Consequently, inflammation markers were not consistently measured before the initiation of tumor-specific therapies. We did not stratify the IPR performance by specific tumor types or stages. Importantly, IPR is not reliable in patients with acute inflammatory conditions, cirrhosis, or nephrotic syndrome—all of which significantly alter serum protein composition. For example, cirrhosis is associated with impaired hepatic albumin synthesis, and nephrotic syndrome leads to urinary loss of albumin and globulins, both of which could result in falsely altered IPR values [48,49]. Similarly, acute inflammation can elevate α-globulin levels, artificially inflating the IPR independent of malignant status. These comorbidities represent important confounders and should be carefully considered when interpreting IPR values. An additional limitation is the absence of TNM staging information for all cancer patients. Finally, the retrospective nature of the study did not allow for the evaluation of the relationship between inflammation monitoring and clinical response to therapy.

Despite these limitations, the IPR demonstrated robust diagnostic characteristics, particularly its notably high NPV, supporting its potential clinical utility as a screening or rule-out test for cancer in complex inpatient populations. However, these findings should be considered preliminary and hypothesis-generating. Prospective studies enrolling patients before the initiation of any tumor-specific therapy, with complete TNM staging and histological characterization, are needed to validate the diagnostic role of the IPR. In addition, the potential use of the IPR as a follow-up biomarker for monitoring therapeutic response and disease progression warrants further investigation.

## 4. Materials and Methods 

### 4.1. Study Design and Population

We retrospectively analyzed data from 612 patients aged ≥18, who were consecutively admitted to the ward of Internal Medicine at the Policlinico Foggia, Italy, from November 2023 to July 2024.

**Figure 3:**
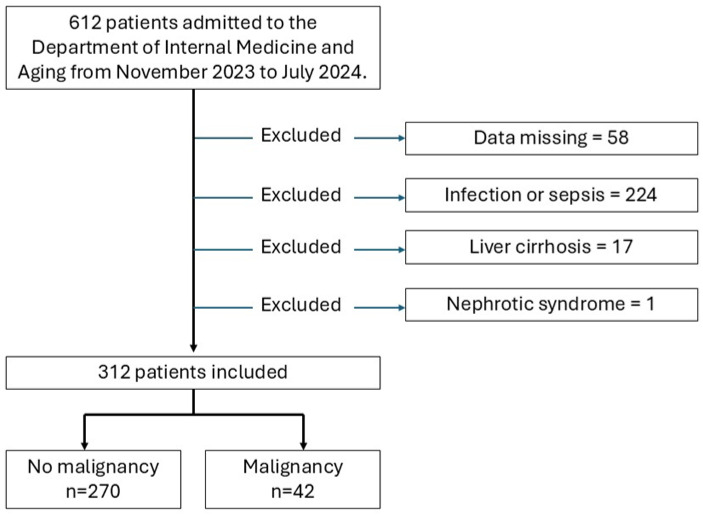
Flowchart of the study population showing included patients and reasons for exclusion.

The exclusion criteria were as follows: incomplete medical records, active infection or sepsis, liver cirrhosis, and nephrotic syndrome. The final study population included 312 patients (Figure 3).

Active cancer was defined based on the National Institute for Health and Care Excellence (NICE) criteria, which include the following: receiving active antimitotic treatment, diagnosed within the last 6 months, recurrent or metastatic, or inoperable cancers [50]. This definition excludes squamous cell carcinoma and basal cell carcinoma.

Patients were divided into two groups based on the presence or absence of active cancer.

Ethical approval for this research was granted by the Institutional Review Board of Policlinico Foggia, and the study was conducted in accordance with the principles outlined in the Declaration of Helsinki.

### 4.2. Clinical Data Collection

Upon admission, a blood sample was collected to measure hemoglobin (Hb), white blood cells (WBC), lymphocytes, serum glucose, albumin, creatinine, and C-reactive protein (CRP), and to perform serum protein electrophoresis (SPE). The neutrophil–lymphocyte ratio (NLR) was calculated by dividing the neutrophil count by the lymphocyte count. The platelet–lymphocyte ratio (PLR) was determined by dividing the platelet count by the lymphocyte count, while the monocyte–lymphocyte ratio (MLR) was calculated as the ratio of monocytes to lymphocytes. The systemic immune–inflammation Iindex (SII) was derived using the following formula: SII = (platelet count × neutrophil count)/lymphocyte count. Additionally, the inflammatory protein Rratio (IPR) was computed as follows, according to Antonucci et al. [20]: IPR = [(*α*1% + *α*2%)/albumin%] × 100.

### 4.3. Statistical Analysis

Data were reported as counts and percentages for categorical variables, and as median and interquartile range (IQR) for continuous variables with a non-normal distribution, or as mean ± standard deviation (SD) for normally distributed continuous variables. The Kolmogorov–Smirnov test was used to assess normality. For group comparisons, the t-test was applied to parametric data, while the Mann–Whitney test was used for non-parametric data. Fisher’s exact test was employed for nominal and categorical variables. The performance of the IPR in identifying frail patients was assessed along with other inflammatory markers (NLR, PLR, MLR, and SII) through receiver operating characteristic (ROC) curve analysis. A *p*-value < 0.05 was considered statistically significant, and all analyses were performed using STATA version 14.

A post hoc power analysis was performed to assess whether the available sample size was adequate to detect a meaningful diagnostic difference in the ability of the IPR to identify active cancer. The analysis was conducted using the power.roc.test() function from the pROC package in R (version 2023.09.1 Build 494), based on the observed area under the ROC curve (AUC = 0.868), 42 cancer cases, and 270 controls. Assuming a significance level of 0.05 and a null hypothesis AUC of 0.5 (no discrimination), the analysis yielded a statistical power close to 1.0.

To assess the added diagnostic value of the IPR compared to conventional inflammatory markers, net reclassification improvement (NRI) and integrated discrimination improvement (IDI) were calculated based on predicted probabilities obtained from univariable logistic regression models including either IPR or a single conventional inflammatory marker as the independent variable.

## 5. Conclusions

In conclusion, our findings indicate that the inflammatory protein ratio (IPR) is a promising and clinically accessible biomarker for the detection of active cancer in hospitalized patients. By integrating alterations in serum proteins modulated by inflammation-driven molecular pathways, the IPR outperformed traditional inflammatory indices in terms of sensitivity, specificity, and overall diagnostic accuracy. While the retrospective and monocentric design of this study represents a limitation, the high negative predictive value supports the potential utility of IPR as a rule-out tool in complex clinical settings. In addition, the IPR relies on routine serum protein electrophoresis—a widely available and low-cost laboratory test—making it easily implementable in real-world hospital workflows. Future prospective, multicenter investigations are needed to validate these findings, explore their generalizability to broader populations, and elucidate the molecular mechanisms linking serum protein shifts to tumor-associated inflammation.

## Figures and Tables

**Table 1:** Type of cancer and prevalence according to WHO classification.

Digestive	14 (4.5%)
Thoracic	10 (3.2%)
Breast	6 (1.9%)
Urinary and Male Genital	4 (1.3%)
Head and Neck	2 (0.6%)
Soft Tissue and Bone	2 (0.6%)
Endocrine	2 (0.6%)
Skin	1 (0.3%)
Female Genital	1 (0.3%)

Data are reported as *n* (%).

**Table 2:** Laboratory parameters of the patients according to the presence or absence of cancer.

	No Malignancy n. 270 (86.5%)	Malignancy n. 42 (13.5%)	*p* Value
Age, years	70.0 (58.0–81.0)	72.5 (66.3–83.5)	0.151
Genre F, *n* (%)	144 (53.3)	19 (45.2)	0.407
Haemoglobin, g/dL	12.6 (11.0–14.0)	11.0 (9.0–12.5)	**<0.001**
WBC, *n*/mm^3^	7085 (5775–9070)	8400 (6472–10732)	**0.006**
Neutrophils, *n*/mm^3^	4354 (3290–5888)	6063 (4112–8418)	**<0.001**
Lymphocytes, *n*/mm^3^	1850 (1345–2295)	1312 (893–1855)	**<0.001**
Monocytes, *n*/mm^3^	497 (383–689)	605 (434–752)	0.087
Platelet, 10^3/mcL	214 (166–266)	251 (167–323)	**0.007**
Albumin, g/dL	3.6 ±0.5	2.8 ±0.5	**<0.001**
Albumin, %	55.8 ±6.3	46.5 ±7.6	**<0.001**
α1 globulin, %	4.0 (3.2–4.8)	5.4 (4.3–7.3)	**<0.001**
α2 globulin, %	10.4 (8.7–12.1)	13.3 (11.9–16.8)	**<0.001**
Creatinine, mg/dL	0.87 (0.73–1.10)	0.78 (0.651.33)	0.893
CRP, ng/mL	3.6 (1.3–15.3)	31.0 (10.7–89.2)	**<0.001**
IPR	25.5 (21.7–31.2)	40.4 (34.5–53.3)	**<0.001**
NLR	2.3 (1.6–3.5)	4.4 (2.2–8.0)	**<0.001**
PLR	119.2 (85.9–169.0)	184.8 (104.9–349.5)	**<0.001**
MLR	0.27 (0.19–0.43)	0.39 (0.29–0.71)	**<0.001**
SII	505 (294–817)	940 (560–2727)	**<0.001**

Sata are expressed as mean (±standard deviation), median (interquartile range), or *n* (percentage) as appropriate. Abbreviations: F, female; CRP, C-reactive protein; IPR, inflammatory protein ratio; NLR, neutrophil-lymphocyte ratio; PLR, platelet-lymphocyte ratio; MLR, monocyte-lymphocyte ratio; SII, systemic immune-inflammation index. *p*-value < 0.05 was considered statistically significant (in bold).

**Table 3:** Diagnostic value of laboratory parameters in identifying the presence of cancer in hospitalized patients.

	AUC (95% CI)	Sensitivity, %	Specificity, %	+LR	−LR	PPV, %	NPV, %	*z* Statistic	*p* Value
IPR	0.868 (0.825–0.903)	88.1	75.2	3.55	0.16	35.7	97.6	14.7	**<0.001**
CRP, ng/mL	0.790 (0.740–0.834)	82.9	68.4	2.63	0.25	29.1	96.3	8.1	**<0.001**
NLR	0.717 (0.664–0.767)	71.4	68.3	2.25	0.42	26.0	93.9	5.0	**<0.001**
PLR	0.707 (0.652–0.757)	64.3	71.4	2.25	0.50	26.0	92.8	4.3	**<0.001**
MLR	0.718 (0.664–0.768)	85.7	50.6	1.73	0.28	21.3	95.8	5.6	**<0.001**
SII	0.730 (0.677–0.779)	71.4	69.7	2.35	0.41	26.9	94.0	5.1	**<0.001**

Abbreviations: AUC, area under the curve; +LR, positive likelihood ratio, −LR, negative likelihood ratio, PPV, positive predictive value; NPV, negative predictive ratio; IPR, inflammatory protein ratio; CRP, C-reactive protein; NLR, neutrophil–lymphocyte ratio; PLR, platelet–lymphocyte ratio; MLR, monocyte–lymphocyte ratio; SII, systemic immune–inflammation index. *p*-value < 0.05 was considered statistically significant (in bold).

**Table 4:** Pairwise comparison of ROC curves for diagnostic performance of IPR versus other inflammatory parameters.

	AUC Difference (95% CI)	*z* Statistic	*p* Value
CRP, ng/mL	0.078 (0.028–0.120)	3.178	**0.001**
NLR	0.151 (0.069–0.241)	3.548	**<0.001**
PLR	0.161 (0.072–0.260)	3.411	**<0.001**
MLR	0.150 (0.083–0.222)	4.320	**<0.001**
SII	0.138 (0.057–0.232)	3.240	**0.001**

The table reports the differences between the AUC of IPR and the AUC of CRP, NLR, PLR, MLR, and SII. Abbreviations: AUC, area under the curve; CRP, C-reactive protein; NLR, neutrophil–lymphocyte ratio; PLR, platelet–lymphocyte ratio; MLR, monocyte–lymphocyte ratio; SII, systemic immune–inflammation index. *p*-value < 0.05 was considered statistically significant (in bold).

## Data Availability

The data presented in this study are available on request from the corresponding author due to ethical and privacy restrictions.

## Abbreviations

The following abbreviations are used in this manuscript:

AUC	Area Under the Curve
CRP	C-reactive Protein
Hb	Hemoglobin
IPR	Inflammatory Protein Ratio
MLR	Monocyte-Lymphocyte Ratio
NLR	Neutrophil-Lymphocyte Ratio
PLR	Platelet-Lymphocyte Ratio
ROC	Receiver Operating Characteristic
SII	Systemic Immune-Inflammation Index
SPE	Serum Protein Electrophoresis
WBC	White Blood Cells
